# Cerebral Aβ deposition precedes reduced cerebrospinal fluid and serum Aβ42/Aβ40 ratios in the *App*^NL−F/NL−F^ knock-in mouse model of Alzheimer’s disease

**DOI:** 10.1186/s13195-023-01196-8

**Published:** 2023-03-25

**Authors:** Emelie Andersson, Nina Schultz, Takashi Saito, Takaomi C. Saido, Kaj Blennow, Gunnar K. Gouras, Henrik Zetterberg, Oskar Hansson

**Affiliations:** 1 Clinical Memory Research Unit, Lund University, 22184 Lund, Sweden; 2 Department of Neurocognitive Science, Institute of Brain Science, Nagoya City University Graduate School of Medical Sciences, Nagoya, Japan; 3 Laboratory for Proteolytic Neuroscience, RIKEN Brain Science Institute, Wako-Shi, Saitama, Japan; 4 Department of Psychiatry and Neurochemistry, Institute of Neuroscience and Physiology, the Sahlgrenska Academy at the University of Gothenburg, Mölndal, Sweden; 5 Clinical Neurochemistry Laboratory, Sahlgrenska University Hospital, Mölndal, Sweden; 6 Department of Experimental Medical Science, Experimental Dementia Research Unit, Lund University, Lund, Sweden; 7 Department of Neurodegenerative Disease, UCL Queen Square Institute of Neurology, Queen Square, London, UK; 8 UK Dementia Research Institute at UCL, London, UK; 9 Hong Kong Center for Neurodegenerative Diseases, Clear Water Bay, Hong Kong, China; 10 Wisconsin Alzheimer’s Disease Research Center, University of Wisconsin School of Medicine and Public Health, University of Wisconsin-Madison, Madison, WI USA; 11 Memory Clinic, SkåneUniversity Hospital, 20502 Malmö, Sweden

**Keywords:** Alzheimer’s disease, Biomarker, Cerebrospinal fluid, Blood, Beta-amyloid

## Abstract

**Background:**

Aβ42/Aβ40 ratios in cerebrospinal fluid (CSF) and blood are reduced in preclinical Alzheimer’s disease (AD), but their temporal and correlative relationship with cerebral Aβ pathology at this early disease stage is not well understood. In the present study, we aim to investigate such relationships using *App* knock-in mouse models of preclinical AD.

**Methods:**

CSF, serum, and brain tissue were collected from 3- to 18-month-old *App*^NL−F/NL−F^ knock-in mice (*n* = 48) and 2–18-month-old *App*^NL/NL^ knock-in mice (*n* = 35). The concentrations of Aβ42 and Aβ40 in CSF and serum were measured using Single molecule array (Simoa) immunoassays. Cerebral Aβ plaque burden was assessed in brain tissue sections by immunohistochemistry and thioflavin S staining. Furthermore, the concentrations of Aβ42 in soluble and insoluble fractions prepared from cortical tissue homogenates were measured using an electrochemiluminescence immunoassay.

**Results:**

In *App*^NL−F/NL−F^ knock-in mice, Aβ42/Aβ40 ratios in CSF and serum were significantly reduced from 12 and 16 months of age, respectively. The initial reduction of these biomarkers coincided with cerebral Aβ pathology, in which a more widespread Aβ plaque burden and increased levels of Aβ42 in the brain were observed from approximately 12 months of age. Accordingly, in the whole study population, Aβ42/Aβ40 ratios in CSF and serum showed a negative hyperbolic association with cerebral Aβ plaque burden as well as the levels of both soluble and insoluble Aβ42 in the brain. These associations tended to be stronger for the measures in CSF compared with serum. In contrast, no alterations in the investigated fluid biomarkers or apparent cerebral Aβ plaque pathology were found in *App*^NL/NL^ knock-in mice during the observation time.

**Conclusions:**

Our findings suggest a temporal sequence of events in *App*^NL−F/NL−F^ knock-in mice, in which initial deposition of Aβ aggregates in the brain is followed by a decline of the Aβ42/Aβ40 ratio in CSF and serum once the cerebral Aβ pathology becomes significant. Our results also indicate that the investigated biomarkers were somewhat more strongly associated with measures of cerebral Aβ pathology when assessed in CSF compared with serum.

**Supplementary Information:**

The online version contains supplementary material available at 10.1186/s13195-023-01196-8.

## Introduction

The presence of aggregated beta-amyloid (Aβ) proteins in the form of extracellular senile plaques in the brain is one of the key neuropathological hallmarks of Alzheimer’s disease (AD) [1]. Aβ derives from sequential cleavage of the transmembrane amyloid precursor protein (APP) by the enzymes β- and γ-secretase [2]. This generates Aβ peptides of different lengths, where the isoform containing 40 amino acids (Aβ40) is the most prevalent while the one containing 42 amino acids (Aβ42) is highly aggregation-prone [3] and found to a large extent in the extracellular senile plaques [4].

Cerebral Aβ pathology can be assessed in vivo by the measurement of the concentration of Aβ42 as well as the Aβ42/Aβ40 ratio in cerebrospinal fluid (CSF), which are well-established fluid biomarkers of AD [5]. The concentration of Aβ42 in CSF is reduced by approximately 50% in patients with AD [6], and longitudinal studies have shown that this change occurs at least a decade before cognitive symptoms manifest, i.e., in the preclinical stage of the disease [7, 8]. In contrast to Aβ42, the concentration of Aβ40 in CSF shows no or minor alteration along the AD continuum [6]. Nevertheless, this peptide can be used to normalize for individual variability in Aβ production, and using the CSF Aβ42/Aβ40 ratio has repeatedly shown to more accurately identify individuals with abnormal cerebral Aβ burden compared with CSF Aβ42 alone [9–13].

The early changes in CSF Aβ42 concentration and the Aβ42/Aβ40 ratio have opened the possibility to identify cognitively healthy individuals who are at high risk of developing dementia due to AD later in life. This has important implications for both clinical management and clinical trials, as disease-modifying therapies probably are most effective when introduced in this early disease stage, before neurodegeneration is widespread [5]. However, the assessment of Aβ in CSF requires invasive lumbar puncture and the alternative use of amyloid positron emission tomography (PET), which is a well-validated imaging biomarker of AD, is expensive and has limited availability. This has led to an intense search for cost-effective and easily accessible blood-based biomarkers that would facilitate biomarker implementation in clinical practice and enable a more efficient screening of potential participants in clinical trials [5, 14]. Indeed, recent development of ultrasensitive immunoassays and high-precision immunoprecipitation mass spectrometry (IP-MS) methods have made it possible to reliably measure Aβ in blood in addition to CSF for the detection of cerebral Aβ pathology in AD [15–19]. The Aβ42/Aβ40 ratio in plasma is highly predictive of cerebral amyloidosis [16, 17, 19] and declines, like the corresponding ratio in CSF, in the preclinical stage of the disease [15, 18, 20]. Indeed, it has been suggested that the Aβ42/Aβ40 ratio in both CSF and plasma may be changed before significant deposition of fibrillar Aβ in the brain, as measured by amyloid PET, is reached [12, 17]. However, it remains uncertain whether these findings reflect different thresholds employed across the techniques used for in vivo measurement of Aβ in fluids and brain or if reduced Aβ42/Aβ40 ratios in CSF and blood serve as indicators of Aβ-related pathological processes that precede the formation of cerebral fibrillar Aβ. Moreover, the ability of the Aβ42/Aβ40 ratio in blood to reflect cerebral Aβ pathology in the brain compared with the corresponding ratio in CSF during the earliest disease stage is not fully elucidated. Further studies are therefore needed to better understand the temporal and correlative relationships between changes in CSF and blood Aβ42/Aβ40 ratios and cerebral Aβ pathology in early preclinical AD.

Although currently available amyloid PET ligands have a high affinity for fibrillar Aβ [21], which is the form of Aβ that dominates in the center of dense-core senile plaques [1], their binding to non-fibrillar plaques is limited [22, 23] and detection of soluble forms of Aβ using this imaging technique is not possible [21]. Thus, to gain a more detailed understanding of how well fluid biomarkers reflect the earliest stages of the Aβ aggregation cascade in AD, animals that recapitulate AD-related pathologies may provide an important model system. A few studies have investigated early changes in CSF and blood Aβ using transgenic mouse models that overexpress mutant human *APP* under the control of certain promoters [24–26]. However, this overexpression paradigm may result in artificial phenotypes that are not related to AD [27], possibly affecting the trajectories of fluid biomarkers in the early pathogenic stage in mice. To overcome this issue, new *App* knock-in mouse models that express endogenous levels of APP while producing pathogenic human Aβ have been introduced to the field [28]. These have in many aspects been shown to better recapitulate AD-related pathologies [27, 28] and may therefore provide a more relevant tool to gain further insight into the dynamics of fluid biomarkers for AD in relation to cerebral Aβ pathology during the early preclinical stage of the disease.

In the present study, we used *App*^NL−F/NL−F^ knock-in mice as a model of preclinical AD to gain further insight into changes in the Aβ42/Aβ40 ratio in CSF and serum and its relation to cerebral Aβ pathology in early stages of the disease. Ultrasensitive single molecule array (Simoa) immunoassays were used to measure the concentrations of Aβ42 and Aβ40 in CSF and serum collected at different time points. Subsequently, we investigated the trajectories of the Aβ42/Aβ40 ratio in the two fluid compartments and their association with different measures of cerebral amyloidosis over time.

## Methods

A graphic overview of the experimental design is presented in Additional file 1: Fig. S1.

### Animals

Experimental procedures were carried out in accordance with Swedish animal research regulations and approved by the committee of animal research at Lund University (ethical permit number: 7482/2017). Animals were housed in groups of 2–6 mice per cage under a 12:12-h light/dark cycle with food and water provided *ad libitum*.

Male and female *App*^NL−F/NL−F^ (3–18 months, *n* = 48) knock-in mice from which paired CSF and serum samples were available were used for the experiments. In these mice, the Aβ sequence of the endogenous *APP* gene has been humanized and two mutations associated with familial AD, the Swedish (KM670/671NL) and Beyreuther/Iberian (I716F), have been introduced. This results in an age-dependent deposition of extracellular amyloid plaques in the cortex and hippocampus starting at 6 months of age [28]. In addition, *App*^NL/NL^ (2–18 months, *n* = 35) knock-in mice that only harbor the Swedish mutation and show no sign of cerebral extracellular plaque deposition during the investigated time period [29] were used as controls.

### Collection of CSF, serum, and brain tissue

All sample collection was performed between 9 AM and 1 PM to minimize the potential influence of the circadian cycle on Aβ40 and Aβ42 concentrations [30].

CSF (around 10 μl) was collected from cisterna magna with a tapered-tip glass capillary as previously described [31]. Following collection, the samples were immediately transferred to protein LoBind tubes, snap frozen on dry ice, and stored at − 80 °C until analysis.

Blood was collected terminally by cardiac puncture, transferred to protein LoBind tubes, allowed to clot for 2 h at room temperature, and centrifuged for 20 min at 2000 × g. The serum supernatant was collected, aliquoted in protein LoBind tubes, and stored at − 80 °C until analysis.

For the collection of brain tissue, transcardial perfusion of the mice with ice-cold 0.1 M phosphate buffer (PB) was performed. The brain was removed and the cortex from the right hemisphere was dissected, collected in protein LoBind tubes, snap frozen on dry ice, and stored at − 80 °C until analysis. The left hemisphere was fixed in 4% paraformaldehyde in 0.1 M PB, pH 7.4, for 48 h at 4 °C and then immersed in 30% sucrose solution for 48 h at 4 °C. Brains were serially cut into 30 μm thick sagittal sections using a sliding microtome (Leica Biosystems) and collected in antifreeze solution (30% sucrose and 30% ethylene glycol in PB) for storage at − 20 °C.

### CSF and serum analysis

The concentrations of human Aβ40 and Aβ42 in CSF and serum were measured using the Simoa Aβ40 and Aβ42 assay kits (Quanterix) on the Simoa HD-1 Analyzer (Quanterix) according to instructions provided by the manufacturer. CSF and serum samples were diluted 1:100 and 1:5, respectively, prior to analysis. The samples were run in singlicates and all measurements were performed in one round of experiment using the same batch of reagents. Intra-assay coefficients of variation, determined using duplicate measurements of internal quality control samples on each plate, were around 5%.

### Histology and immunohistochemistry

30 µm thick free-floating sagittal brain sections were washed 3 × 10 min in TBS, treated 8 min with 88% formic acid (FA), permeabilized 3 × 10 min in TBS containing 0.25% Triton X-100 (TBSX), and blocked 1 h in TBSX containing 5% normal donkey serum (NDS). The sections were then incubated with anti-Aβ42 primary antibody (H31L21, Invitrogen) diluted 1:1000 in TBSX containing 2.5% NDS overnight at 4 °C. Following overnight incubation, the sections were washed 3 × 10 min in TBSX, incubated with appropriate Alexa-fluorophore-conjugated secondary antibody diluted 1:500 in TBSX containing 2.5% NDS, washed 3 × 10 min in TBSX, mounted on glass slides, and coverslipped with ProLong™ Diamond Antifade Mountant (Invitrogen) according to the recommendations from the manufacturer.

For the detection of fibrillar dense-core plaques, free-floating sagittal sections were stained with 0.01% thioflavin S in 50% ethanol for 10 min and then washed 2 × 1 min in 50% ethanol, 3 × 1 min in ddH_2_O, and finally 10 min in TBS. The stained specimens were mounted on glass slides and coverslipped with SlowFade™ Diamond Antifade Mountant (Invitrogen) according to the manufacturer’s recommendations.

### Image acquisition and analysis

Fluorescence images of whole brain sections were acquired using a 10 × objective lens on the Operetta® CLS™ High Content Analysis System (PerkinElmer). The cortex and hippocampus from 3–4 brain sections per mouse were manually segmented and the area covered by Aβ42-positive staining, as well as thioflavin S-positive fibrillar dense-core plaques, was quantified using the Fiji software by applying an automated local threshold that was maintained for all images analyzed. For each mouse, the total cortical and hippocampal area (%) covered was determined by calculating the average of all captured sections.

### Brain tissue homogenization

The cortex from the right hemisphere was homogenized at 10% (w/v) in TBS (50 mM Tris–HCl, 150 mM NaCl, pH 7.6) containing Halt™ Protease and Phosphatase Inhibitor Cocktail (Thermo Fisher Scientific) using the FastPrep-24™ Classic bead beating grinder and lysis system (MP Biomedicals). The homogenized cortical tissue was aliquoted in protein LoBind tubes and stored at − 80 °C until analysis.

For extraction of Aβ, the prepared homogenates were thawed on ice and centrifuged at 14,000 × g for 30 min at 4 °C. The supernatant was collected as the TBS-soluble fraction, aliquoted in protein LoBind tubes, and stored at − 80 °C until analysis. The remaining pellet was re-suspended at 10% (v/w) in ice-cold 70% FA containing Halt™ Protease and Phosphatase Inhibitor Cocktail, sonicated on ice for 6 × 10 s, and centrifuged at 14,000 × g for 1 h at 4 °C. The supernatant was collected as the FA-soluble fraction, neutralized 1:20 in 1 M Tris-base at room temperature, aliquoted in protein LoBind tubes, and stored at − 80 °C until analysis.

### Biochemical analysis of Aβ42 in soluble and insoluble brain tissue extracts

The concentration of Aβ42 in the TBS- and FA-soluble fractions prepared from cortical brain tissue homogenates was measured using the MSD V-PLEX Human Aβ42 Peptide (6E10) Kit according to the manufacturer’s recommendations. Samples from the TBS-soluble fraction were diluted 1:16, whereas those from the FA-soluble fraction were diluted up to 1:640. All samples were measured in singlicates, as this assay consistently has shown a low intra-plate coefficient of variation in previous analyses.

### Statistical analysis

The nonparametric Kruskal–Wallis *H* test was performed to study age-dependent changes of Aβ in the CSF, serum, and brain. If a statistical significance was found, *post hoc* analyses for group comparisons between all groups were performed using the Mann–Whitney *U* test. Correlation analyses were done using Spearman’s rank-ordered correlation coefficient. For comparisons between correlation coefficients, Meng’s *Z*-test for correlated correlations was performed [32]. Statistical analyses were conducted using IBM SPSS Statistics 27 and corresponding graphs were produced in GraphPad Prism 9.

## Results

### The Aβ42/Aβ40 ratio in CSF was reduced from 12 months of age in *App*^NL−F/NL−F^ knock-in mice

**Fig. 1:**
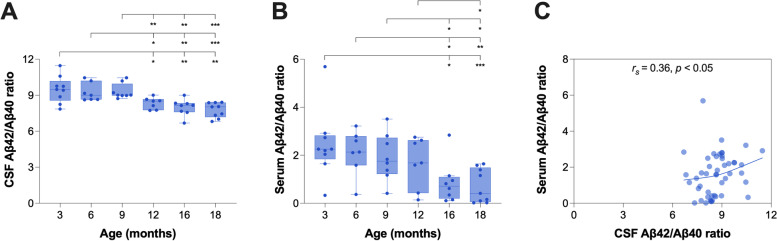
CSF and serum Aβ42/Aβ40 ratios in *App*^NL−F/NL−F^ knock-in mice. CSF and serum Aβ42/Aβ40 ratios were measured in 3 (*n* = 9)-, 6 (*n* = 7)-, 9 (*n* = 8)-, 12 (*n* = 7)-, 16 (*n* = 8)-, and 18 (*n* = 9)-month-old *App*^NL−F/NL−F^ knock-in mice. **A** The Aβ42/Aβ40 ratio in CSF showed a significant reduction from 12 months of age while **B** the  Aβ42/Aβ40 ratio in serum was significantly declined from 16 months of age. **C** The Aβ42/Aβ40 ratio in serum correlated significantly positive with the corresponding ratio in CSF. Data is presented as median and IQR. Whiskers represent data within 1.5IQR of the lower and upper quartiles. For comparison between groups, statistical analyses were performed using the Kruskal–Wallis *H* test followed by the Mann–Whitney *U* test for *post hoc* group comparisons (*p < 0.05, **p < 0.01, ***p < 0.001). Correlation analysis was performed using Spearman’s rank-ordered correlation coefficient. Abbreviations: Aβ, amyloid beta; CSF, cerebrospinal fluid; IQR, interquartile range

In CSF collected from *App*^NL−F/NL−F^ knock-in mice, the Aβ42/Aβ40 ratio was significantly affected by age (*H*(5) = 30.9, *p* < 0.001), in which pairwise comparisons revealed a lower Aβ42/Aβ40 ratio from 12 months (Fig. 1A). Similar results were obtained for CSF Aβ42 (*H*(5) = 30.8, *p* < 0.001), while no significant change in CSF Aβ40 was observed (*H*(5) = 9.4, *p* > 0.05) (Additional file 1: Fig. S2A-B). In *App*^NL/NL^ knock-in mice, some fluctuations in the CSF Aβ42/Aβ40 ratio were found over time (*H*(4) = 15.9, *p* < 0.01), although age did not significantly affect the concentrations of CSF Aβ42 (*H*(4) = 2.0, *p* > 0.05) or Aβ40 (*H*(4) = 6.3, *p* > 0.05) (Additional file 1: Fig. S3A-C).

### The Aβ42/Aβ40 ratio in serum was reduced from 16 months of age and correlated with the corresponding ratio in CSF in *App*^NL−F/NL−F^ knock-in mice

There was a significant age-dependent effect on the serum Aβ42/Aβ40 ratio in *App*^NL−F/NL−F^ knock-in mice (*H*(5) = 16.5, *p* < 0.01), in which pairwise comparisons revealed a lower Aβ42/Aβ40 ratio from 16 months (Fig. 1B). Similar results were found for serum Aβ42 (*H*(5) = 12.4, *p* < 0.05), while serum Aβ40 was significantly increased from 16 months (*H*(5) = 11.3, *p* < 0.05) (Additional file 1: Fig. S2C-D). In *App*^NL/NL^ knock-in mice, no significant age-dependent effect on the serum Aβ42/Aβ40 ratio (*H*(4) = 3.3, *p* > 0.05), Aβ42 (*H*(4) = 0.8, *p* > 0.05), or Aβ40 (*H*(4) = 6.5, *p* > 0.05) was observed (Additional file 1: Fig. S3D-F).

There was a significant, but moderate, positive correlation between serum and CSF Aβ42/Aβ40 ratios in *App*^NL−F/NL−F^ knock-in mice when measured over all age groups (*r*_s_ = 0.36, *p* < 0.05) (Fig. 1C). A similar correlation was obtained between serum and CSF Aβ42 (*r*_s_ = 0.33, *p* < 0.05), while no correlation for Aβ40 was found (*r*_s_ =  − 0.11, *p* > 0.05) (Additional file 1: Fig. S2E-F). In *App*^NL/NL^ knock-in mice, there was no correlation between serum and CSF Aβ42/Aβ40 ratios (*r*_s_ = 0.20, *p* > 0.05), Aβ42 (*r*_s_ =  − 0.27, *p* > 0.05), or Aβ40 (*r*_s_ =  − 0.047, *p* > 0.05) (Additional file 1: Fig. S3G-I).

### Extracellular amyloid plaques were increased in an age-dependent manner and inversely correlated with CSF and serum Aβ42/Aβ40 ratios in *App*^NL-F/NL-F^ knock-in mice

**Fig. 2:**
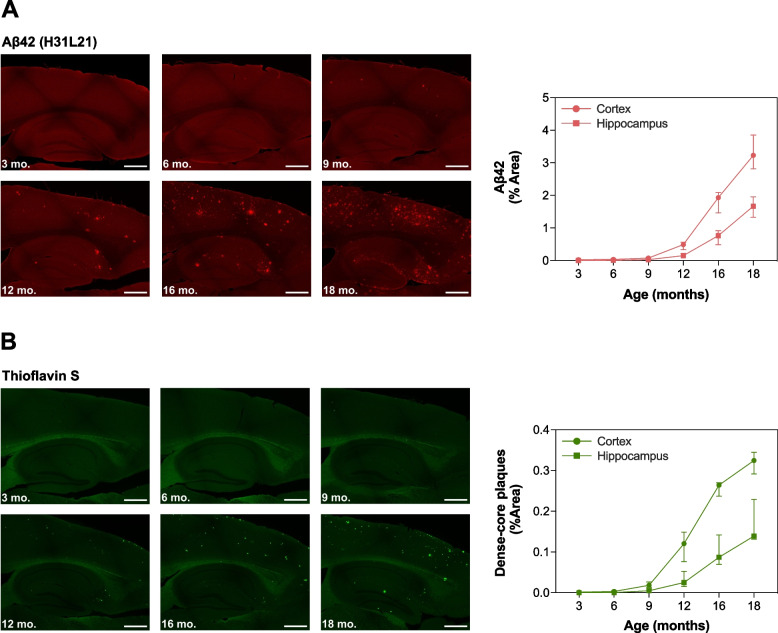
Cerebral Aβ plaque burden in *App*^NL−F/NL−F^ knock-in mice. Cerebral Aβ42 immunoreactivity and thioflavin S-positive fibrillar dense-core plaques were measured in 3 (*n* = 9)-, 6 (*n* = 7)-, 9 (*n* = 8)-, 12 (*n* = 7)-, 16 (*n* = 8)-, and 18 (*n* = 9)-month-old *App*^NL−F/NL−F^ knock-in mice. Minor initial deposition of extracellular Aβ plaques in cortical brain regions was observed from 6 months of age. The burden of cortical and hippocampal **A** Aβ42 immunoreactivity and **B** thioflavin S-positive fibrillar dense-core plaques was significantly increased in an age-dependent manner. Data is presented as median and IQR. Whiskers represent data within 1.5IQR of the lower and upper quartiles. For comparison between groups, statistical analyses were performed using the Kruskal–Wallis *H* test followed by the Mann–Whitney *U* test for *post hoc* group comparisons. Scale bars: 500 μm. Abbreviations: Aβ, amyloid beta; IQR, interquartile range

Immunohistochemical analysis revealed very minor initial deposition of extracellular amyloid plaques in cortical brain regions at 6 months of age in *App*^NL−F/NL−F^ knock-in mice. The burden of cortical and hippocampal Aβ42 immunoreactivity was increased in an age-dependent manner (*H*(5)_Cortex_ = 51.0, *p* < 0.001; *H*(5)_Hippocampus_ = 48.4, *p* < 0.001), in which it became more widespread from approximately 12 months of age (Fig. 2A and Additional file 1: Table S1). Evaluation of the burden of thioflavin S-positive fibrillar dense-core plaques in the two regions showed similar results with clear, but still rather modest, increases in plaque burden at 12 months of age (*H*(5)_Cortex_ = 52.1, *p* < 0.001; *H*(5)_Hippocampus_ = 47.7, *p* < 0.001) (Fig. 2B and Additional file 1: Table S1). No deposition of extracellular amyloid plaques was found in *App*^NL/NL^ knock-in mice over time (Additional file 1: Fig. S3J).

**Fig. 3:**
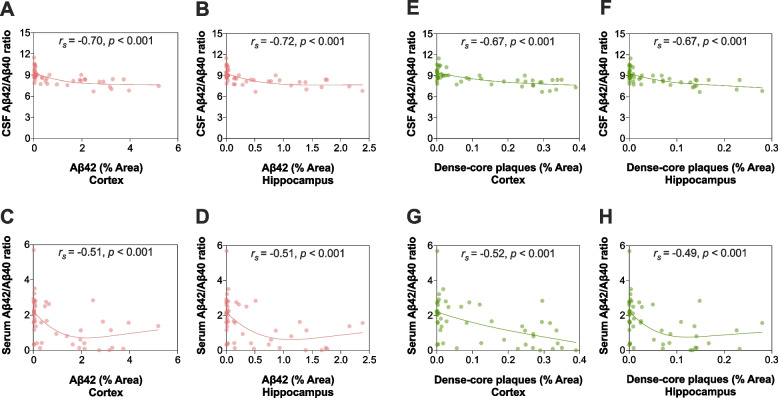
CSF and serum Aβ42/Aβ40 ratios and their associations with cerebral Aβ plaque burden in *App*^NL−F/NL−F^ knock-in mice. In the whole study population, the Aβ42/Aβ40 ratio in CSF and serum inversely correlated with **A**–**D** Aβ42 immunoreactivity and **E**–**H** thioflavin S-positive fibrillar dense-core plaques in the cortex and hippocampus. Correlation analyses were performed using Spearman’s rank-ordered correlation coefficient. Abbreviations: Aβ, amyloid beta; CSF, cerebrospinal fluid

In the whole study population, the Aβ42/Aβ40 ratio in both CSF and serum showed a significant inverse correlation with Aβ42 immunoreactivity in the cortex (*r*_s(CSF Aβ42/Aβ40 ratio)_ =  − 0.70, *p* < 0.001; *r*_s(serum Aβ42/Aβ40 ratio)_ =  − 0.51, *p* < 0.001) and hippocampus (*r*_s(CSF Aβ42/Aβ40 ratio)_ =  − 0.72, *p* < 0.001; *r*_s(serum Aβ42/Aβ40 ratio)_ =  − 0.51, *p* < 0.001) (Fig. 3A–D). These associations appeared hyperbolic, in which the Aβ42/Aβ40 ratio in CSF and serum stabilized toward a plateau while Aβ42 immunoreactivity steadily continued to increase as the mice age. Similar results were found when evaluating the correlations between the fluid biomarkers and the burden of thioflavin S-positive fibrillar dense-core plaques in the cortex (*r*_s(CSF Aβ42/Aβ40 ratio)_ =  − 0.67, *p* < 0.001; *r*_s(serum Aβ42/Aβ40 ratio)_ =  − 0.52, *p* < 0.001) and hippocampus (*r*_s(CSF Aβ42/Aβ40 ratio)_ =  − 0.67, *p* < 0.001; *r*_s(serum Aβ42/Aβ40 ratio)_ =  − 0.49, *p* < 0.001) (Fig. 3E–H). Correlations similar to those shown for the Aβ42/Aβ40 ratio were obtained for CSF and serum Aβ42, but not for Aβ40 (Additional file 1: Fig. S4).

The correlations between the Aβ42/Aβ40 ratio in the two fluid compartments and pathological changes of cerebral plaque burden (measured by Aβ42 immunoreactivity and the burden of thioflavin S-positive fibrillar dense-core plaques) were numerically greater when the Aβ42/Aβ40 ratio was measured in CSF compared with serum, but the differences in correlation coefficients were not statistically significant (Table 1). Similar findings were obtained for Aβ42, although the concentrations in CSF were significantly more strongly associated with plaque burden in the hippocampus than those in serum (Additional file 1: Table S2).

### Soluble and insoluble Aβ42 in cortical brain tissue were increased in an age-dependent manner and inversely correlated with CSF and serum Aβ42/Aβ40 ratios in *App*^NL-F/NL-F^ knock-in mice

**Fig. 4:**
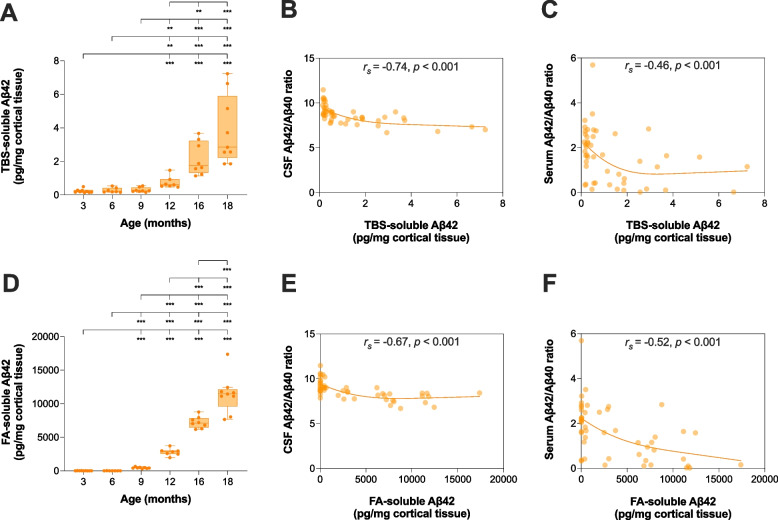
Cortical TBS- and FA-soluble Aβ42 and their associations with CSF and serum Aβ42/Aβ40 ratios in *App*^NL−F/NL−F^ knock-in mice. Cortical TBS- and FA-soluble Aβ42 was measured in 3 (*n* = 9)-, 6 (*n* = 7)-, 9 (*n* = 8)-, 12 (*n* = 7)-, 16 (*n* = 8)-, and 18 (*n* = 9)-month-old *App*^NL−F/NL−F^ knock-in mice. **A** TBS-soluble and **D** FA-soluble Aβ42 increased in an age-dependent manner with significant changes from 12 and 9 months, respectively. In the whole study population, the Aβ42/Aβ40 ratio in CSF and serum inversely correlated with Aβ42 in the **B-C **TBS-soluble fraction and the **E-F **FA-soluble fraction prepared from cortical brain tissue homogenates. Data is presented as median and IQR. Whiskers represent data within 1.5IQR of the lower and upper quartiles. For comparison between groups, statistical analyses were performed using the Kruskal–Wallis *H* test followed by the Mann–Whitney *U* test for *post hoc* group comparisons (**p < 0.01, ***p < 0.001). Correlation analyses were performed using Spearman’s rank-ordered correlation coefficient. Abbreviations: Aβ, amyloid beta; CSF, cerebrospinal fluid; FA, formic acid; IQR, interquartile range; TBS, tris-buffered saline

In the TBS- and FA-soluble fractions prepared from cortical brain tissue homogenates, the concentration of Aβ42 was increased in an age-dependent manner, with significant alterations from 12 and 9 months, respectively (*H*(5)_TBS-soluble_ = 38.4, *p* < 0.001; *H*(5)_FA-soluble_ = 44.1, *p* < 0.001) (Fig. 4A, D). The Aβ42/Aβ40 ratio in CSF and serum inversely correlated with the concentration of Aβ42 in the TBS-soluble fraction (*r*_s(CSF Aβ42/Aβ40 ratio)_ =  − 0.74, *p* < 0.001; *r*_s(serum Aβ42/Aβ40 ratio)_ =  − 0.46, *p* < 0.001) (Fig. 4B-C), as well as in the FA-soluble fraction (*r*_s(CSF Aβ42/Aβ40 ratio)_ =  − 0.67, *p* < 0.001; *r*_s(serum Aβ42/Aβ40 ratio)_ =  − 0.52, *p* < 0.001) (Fig. 4E-F), in a hyperbolic manner. Similar results as shown for the Aβ42/Aβ40 ratios were obtained for CSF and serum Aβ42, but not for Aβ40 (Additional file 1: Fig. S5).

**Table 1:** Correlations between the Aβ42/Aβ40 ratio in CSF and serum and cerebral Aβ pathology in *App*^NL−F/NL−F^ knock-in mice

	**CSF Aβ42/Aβ40 ratio**	**Serum Aβ42/Aβ40 ratio**	**Meng’s *Z*-test (*p*-value)**
Cortical Aβ42 (%Area)	*r*_s_ = − 0.70	*r*_s_ = − 0.51	0.11
Hippocampal Aβ42 (%Area)	*r*_s_ = − 0.72	*r*_s_ = − 0.51	0.073
Cortical dense-core plaques (%Area)	*r*_s_ = − 0.67	*r*_s_ = − 0.52	0.22
Hippocampal dense-core plaques (%Area)	*r*_s_ = − 0.67	*r*_s_ = − 0.49	0.15
TBS-soluble Aβ42 (pg/mg cortical tissue)	***r***_**s**_** = ** − **0.74**	***r***_**s**_** = ** − **0.46**	**0.018**
FA-soluble Aβ42 (pg/mg cortical tissue)	*r*_s_ = − 0.67	*r*_s_ = − 0.52	0.22

Correlation analyses were performed using Spearman’s rank-ordered correlation coefficient. Differences between correlation coefficients were estimated using Meng’s *Z*-test

*Abbreviations*: *Aβ* amyloid beta, *CSF* cerebrospinal fluid, *FA* formic acid, *TBS* tris-buffered saline

The correlation with TBS-soluble Aβ42 was significantly greater for the Aβ42/Aβ40 ratio when measured in CSF compared with serum. However, although the Aβ42/Aβ40 ratio in CSF showed a greater correlation with FA-soluble Aβ42 than the corresponding ratio in serum, these were not statistically different (Table 1). Similar findings were obtained for Aβ42 when measured in CSF compared with serum (Additional file 1: Table S2).

## Discussion

In the present study, our main findings showed that the Aβ42/Aβ40 ratio in both CSF and serum were reduced during early cerebral amyloidosis in *App*^NL−F/NL−F^ knock-in mice. Significant changes in the CSF Aβ42/Aβ40 ratio occurred when cerebral Aβ plaque burden started to become more widespread in cortical and hippocampal regions, while the corresponding ratio in serum was altered at a somewhat later time point. Furthermore, the initial decline of the CSF Aβ42/Aβ40 ratio coincided with increased concentrations of soluble and insoluble Aβ42 in cortical brain tissue. In both fluid compartments, the reduction in the Aβ42/Aβ40 ratio quickly started to stabilize towards a plateau although both insoluble and soluble forms of Aβ steadily continued to increase as the mice aged. Accordingly, we found inverse hyperbolic associations between cerebral Aβ and the Aβ42/Aβ40 ratio in both CSF and serum. These associations tended to be greater for the measures in CSF compared with serum. In general, similar results were obtained for CSF and serum Aβ42, but not Aβ40, when compared to those obtained for the Aβ42/Aβ40 ratio.

### The Aβ42/Aβ40 ratio in CSF was significantly reduced earlier than in blood in *App*^NL-F/NL-F^ knock-in mice

The Aβ42/Aβ40 ratio in human CSF declines at least a decade before cognitive symptoms due to both sporadic and familial AD develop [7, 8], and recent studies using highly sensitive biochemical assays suggest that the corresponding measure in plasma also is reduced during preclinical sporadic AD [15, 18, 20]. Our findings in the *App*^NL−F/NL−F^ knock-in mice are in good agreement with these studies, demonstrating an age-dependent decline in CSF and serum Aβ42/Aβ40 ratios, changes that occur prior to the time at which Aβ-dependent spatial memory deficits develop in these mice [28]. The Aβ42/Aβ40 ratio in CSF was steadily reduced from 12 months of age, while the corresponding ratio in serum significantly declined somewhat later, at 16 months of age. The decline in serum Aβ42/Aβ40 ratio was partially due to elevated concentrations of Aβ40 in the oldest age groups. This is in contrast to previous studies in sporadic AD, in which the concentrations of this Aβ peptide have been reported to remain unaltered during the preclinical stage of the disease [15, 18]. Nevertheless, considering that the increase in serum Aβ40 coincided with the decrease in serum Aβ42 and that we found no age-dependent effect on serum Aβ40 in control *App*^NL/NL^ knock-in mice, it is possible that the concentrations of Aβ40 are elevated in response to AD-related pathological processes in *App*^NL−F/NL−F^ knock-in mice. Future studies should address the potential impact of the Beyreuther/Iberian mutation and the relevance of this finding for sporadic and familial AD.

In *App*^NL−F/NL−F^ knock-in mice, the Aβ42/Aβ40 ratio in serum was positively correlated with that in CSF when assessed over all age groups, although the association was relatively modest. These results are well in line with previous findings in which the Simoa platform was used to measure plasma Aβ42 and Aβ40 in a clinical cohort consisting of cognitively healthy individuals as well as patients with mild cognitive impairment (MCI) and AD dementia [15]. The modest association may to some extent be explained by physiological confounding factors that influence the measurement of Aβ in blood compared with CSF, such as degradation in the liver or by circulating enzymes, matrix effects, and renal clearance [33]. Moreover, it is likely that the production of these peptides in peripheral organs significantly contributes to the circulating pool, as it has been estimated that a maximum of 30–50% of Aβ present in blood derives from the central nervous system [34]. The choice of analytical platform may also play a role, as the use of methods based on IP-MS has recently been reported to generate higher associations between the Aβ42/Aβ40 ratio in the two fluid compartments compared to different Simoa immunoassays [35].

### CSF and serum Aβ42/Aβ40 ratios in relation to cerebral amyloidosis

In agreement with previous findings [28], a few isolated extracellular Aβ plaques first appeared in cortical areas of the brain at 6 months of age in *App*^NL−F/NL−F^ knock-in mice. At 9 months, the cerebral Aβ plaque burden remained sparse and did not associate with changes in the Aβ42/Aβ40 ratio in either CSF or serum. Instead, the decline of this biomarker in the two fluid compartments at 12 and 16 months, respectively, occurred in relation to a more pronounced Aβ plaque load in both cortex and hippocampus. In a recent study in autopsy-confirmed AD cases, the authors reported that a decline in the CSF Aβ42/Aβ40 ratio was initiated in Thal phase 2 [36], which is characterized by the presence of Aβ deposits in neocortex and allocortical regions [37]. No changes in CSF Aβ42/Aβ40 ratio were found in cases in which the pathology was restricted to the neocortex, *i.e.*, in Thal phase 1 [36]. The study also showed that a reduced CSF Aβ42/Aβ40 ratio was associated with a moderate Aβ plaque burden, as estimated in accordance with CERAD (Consortium to Establish a Registry for Alzheimer’s Disease) recommendations, a finding that is similar to what has been observed for the Aβ42/Aβ40 ratio in plasma [38]. These results are in line with those from the present study, suggesting a temporal sequence of events in which initial deposition of Aβ aggregates in restricted brain regions is followed by a decline in CSF and serum Aβ42/Aβ40 ratios once the Aβ pathology is somewhat more widespread but still relatively low to moderate. In addition, studies have suggested that CSF Aβ42—alone or in ratio with Aβ40 [12, 39–42]—as well as the Aβ42/Aβ40 ratio in plasma [17] are significantly changed before the threshold for abnormal fibrillar dense-core plaque burden in the brain, as measured by amyloid PET, is reached. It is possible that the sensitivity of amyloid PET to detect fibrillar Aβ species in the brain in early preclinical AD is limited, as the results from the present study suggest a sparse burden of thioflavin S-positive fibrillar dense-core plaques in the brain prior to changes in the investigated fluid biomarkers.

Insoluble forms of Aβ found predominantly in plaques can be measured biochemically in FA extract from brain tissue homogenates. As expected, cortical FA-soluble Aβ42 increased in an age-dependent manner with significant changes from 9 months of age, which is the same time from which we also observed a sparse burden of cortical Aβ plaques in *App*^NL−F/NL−F^ knock-in mice. In addition, cortical TBS-soluble Aβ42 increased from 12 months of age and thereby coincided with the initial decline in the CSF Aβ42/Aβ40 ratio. Although both insoluble and soluble forms of Aβ steadily increased over the studied time period, the reduced Aβ42/Aβ40 ratio in both CSF and serum quickly started to stabilize toward a plateau. Indeed, we found an inverse hyperbolic association between cerebral amyloidosis and the Aβ42/Aβ40 ratio in both CSF and serum, which is consistent with multiple cross-sectional studies in humans investigating the association between Aβ42—alone or in ratio with Aβ40—in the two fluid compartments and amyloid PET [9, 12, 17, 43–45]. Our results are also in good agreement with longitudinal studies suggesting that once a decline in CSF Aβ42 has occurred in early preclinical AD, the concentration remains fairly stable as the disease progresses [8, 46, 47]. Together, these findings may to some extent challenge the proposed hypothesis that the reduction in the investigated fluid biomarkers is due to the deposition of Aβ into extracellular plaques [48], as cerebral plaque load is only linearly associated with the Aβ42/Aβ40 ratio in CSF and blood during a very limited time-frame in the early disease stage. However, our findings that no changes in CSF or serum Aβ were found in *App*^NL/NL^ mice with age confirm that biological processes related to Aβ pathology are required for these biomarkers to decline. Future studies should further address the underlying cause of these changes in the preclinical stage of AD.

The inverse correlation with cerebral amyloidosis tended to be greater for the Aβ42/Aβ40 ratio in CSF when compared with serum. These results imply that the Aβ42/Aβ40 ratio in CSF more reliably may reflect Aβ pathology in the brain than the corresponding ratio in blood in preclinical AD. In agreement with these findings, a study conducted by Schindler *et al*. reported that the Aβ42/Aβ40 ratio in CSF was a better predictor of and showed a greater correlation with amyloid PET than the Aβ42/Aβ40 ratio in plasma in a cohort consisting of mainly cognitively healthy individuals [17]. Furthermore, although the ratio between plasma Aβ42 and Aβ40 has shown higher correspondence with cerebral amyloidosis than Aβ42 alone when studied in clinical cohorts [16], the correlations between cerebral amyloidosis and these two measures in serum were similar in *App*^NL−F/NL−F^ knock-in mice. As the concentrations of Aβ42 and Aβ40 in blood may be affected by comorbidities and other confounding factors [15, 49], the limited biological variation in the *App*^NL−F/NL−F^ knock-in mice compared to a human study population may to some extent explain these results.

A few studies have previously investigated Aβ changes in CSF [24, 25] and blood [26] in relation to cerebral amyloidosis over time using transgenic mouse models that overexpress mutant human *APP* under the control of certain promotors. In line with our own findings in *App*^NL−F/NL−F^ knock-in mice, these studies have reported a decline in Aβ42—alone or in ratio with CSF Aβ40—in these fluid compartments that is initiated shortly after the onset of Aβ plaque deposition and inversely associates with the burden of Aβ in the brain. In one of the studies, increased concentrations of both CSF Aβ42 and Aβ40 prior to plaque deposition were observed, suggesting that the biphasic profile of this biomarker change potentially could be used for early identification of cognitively healthy individuals who are at risk of developing AD dementia [25]. Although we have observed similar findings in the well-characterized *APP*-overexpressing 3xTg mouse model (Additional file 1: Fig. S6 and Supplementary methods), this initial increase was not found in *App*^NL−F/NL−F^ knock-in mice. The concomitant increase in CSF Aβ42 and Aβ40 in *APP*-overexpressing mice suggests an elevated production or cleavage of APP and one may speculate that this early change to some extent is a result of an age-related overexpression of *APP* in these models. However, further studies are needed to elucidate these differences and their translational implication.

### Limitations

A limitation of the study was the use of the Simoa platform for measurements of Aβ42 and Aβ40 in serum. Namely, it was recently demonstrated that the Aβ42/Aβ40 ratio in plasma more accurately identifies individuals with an abnormal burden of cerebral Aβ when assessed with certain methods based on IP-MS compared with Simoa immunoassays [35]. However, we could not sample enough volumes of serum from the mice to perform IP-MS. In addition, assessment of Aβ42 and Aβ40 in plasma instead of serum would have been preferable from a translational perspective, as plasma has been most commonly analyzed in previous clinical studies. Furthermore, we were not able to measure the concentrations of Aβ40 in TBS- and FA-soluble cortical extracts from *App*^NL−F/NL−F^ knock-in mice in the younger age groups with our current protocol, likely as a result of the low concentrations of this Aβ peptide due to the Beyreuther/Iberian mutation.

## Conclusion

Taken together, our findings suggest that a low burden of cerebral Aβ pathology may be present before the Aβ42/Aβ40 ratio in CSF and serum starts to decline. These changes in fluid Aβ42/Aβ40 ratios seem to occur in association with a more widespread Aβ plaque burden in cortical and hippocampal regions, which occurs in parallel with increasing concentrations of insoluble and soluble Aβ42 in the brains of the *App*^NL−F/NL−F^ knock-in mice. The Aβ42/Aβ40 ratio in CSF seems to decline prior to the corresponding ratio in serum and may be the most reliable biomarker of early cerebral Aβ pathology of the two measures. Our findings raise the possibility that previous reports from human studies showing that an abnormal deposition of Aβ in the brain, as determined by amyloid PET, is reached after fluid Aβ42/Aβ40 ratios decline may be due to the limited sensitivity of this imaging technique to detect such lesions in the early preclinical phase of AD. This extends existing knowledge of the temporal relationship between early cerebral Aβ pathology and initial changes in fluid Aβ42/Aβ40 ratios and may have implications for the use of these biomarkers in future human studies aiming at better understanding the disease. Moreover, as disease-modifying therapies are likely to be more effective the earlier in the pathological process they are introduced, our results indicate the need for further research to identify fluid biomarkers reflecting the initial amyloidogenic phase of the disease. In this context, *App* knock-in mice, which possess less artifacts and may in many aspects more accurately recapitulate Aβ-related pathological processes in AD compared with first-generation *APP*-overexpressing transgenic mice, could provide a valuable translational tool. The use of such models may also contribute to important information on the underlying cause of changes in CSF biomarkers of Aβ pathology in preclinical AD and how these are affected by disease-modifying therapies.

## Supplementary Information

**Additional file 1.** Supplementary information.

## Data Availability

The datasets used and/or analyzed during the current study are available from the corresponding authors on reasonable request.

## Abbreviations

Aβ: Beta-amyloid
Aβ40: 40 Amino acid beta-amyloid peptide
Aβ42: 42 Amino acid beta-amyloid peptide
AD: Alzheimer’s disease
APP: Amyloid precursor protein
CERAD: Consortium to Establish a Registry for Alzheimer’s Disease
CSF: Cerebrospinal fluid
FA: Formic acid
IP-MS: Immunoprecipitation mass spectrometry
MCI: Mild cognitive impairment
MSD: Meso Scale Discovery
NDS: Normal donkey serum
PB: Phosphate buffer
PET: Positron emission tomography
Simoa: Single molecule array
TBS: Tris-buffered saline
TBSX: Tris-buffered saline with Triton X-100
