# Supplementary material for: Cerebral Aβ deposition precedes reduced cerebrospinal fluid and serum Aβ42/Aβ40 ratios in the AppNL−F/NL−F knock-in mouse model of Alzheimer’s disease
Source: Alzheimers Res Ther. 2023 Mar 25;15:64. doi: 10.1186/s13195-023-01196-8 (PMC10039589; doi:10.1186/s13195-023-01196-8)
Supplement: Supplementary file 1 — Additional file 1. Supplementary information. [file 13195_2023_1196_MOESM1_ESM.pdf]

Supplementary information

Cerebral Aβ deposition precedes reduced cerebrospinal fluid and serum Aβ42/Aβ40 ratios in the *App*$^{NL-F/NL-F}$ knock-in mouse model of Alzheimer's disease

Emelie Andersson[1], Nina Schultz[1], Takashi Saito[2], Takaomi C. Saido[3], Kaj Blennow[4, 5], Gunnar K. Gouras[6], Henrik Zetterberg[4, 5, 7, 8, 9, 10], Oskar Hansson[1, 11]

[1] Clinical Memory Research Unit, Lund University, Lund, Sweden

[2] Department of Neurocognitive Science, Institute of Brain Science, Nagoya City University Graduate School of Medical Sciences, Nagoya, Japan

[3] Laboratory for Proteolytic Neuroscience, RIKEN Brain Science Institute, Wako-shi, Saitama, Japan

[4] Department of Psychiatry and Neurochemistry, Institute of Neuroscience and Physiology, the Sahlgrenska Academy at the University of Gothenburg, Mölndal, Sweden

[5] Clinical Neurochemistry Laboratory, Sahlgrenska University Hospital, Mölndal, Sweden

[6] Experimental Dementia Research Unit, Lund University, Lund, Sweden.

[7] Department of Neurodegenerative Disease, UCL Queen Square Institute of Neurology, Queen Square, London, UK

[8] UK Dementia Research Institute at UCL, London, UK

[9] Hong Kong Center for Neurodegenerative Diseases, Clear Water Bay, Hong Kong, China

[10] Wisconsin Alzheimer's Disease Research Center, University of Wisconsin School of Medicine and Public Health, University of Wisconsin-Madison, Madison, WI, USA

[11] Memory Clinic, Skåne University Hospital, SE-20502 Malmö, Sweden

Corresponding authors

Emelie Andersson, Clinical Memory Research Unit, Lund University, SE-22184 Lund, Sweden, emelie.andersson@med.lu.se, phone +46 46 222 96 67

Oskar Hansson, Memory Clinic, Skåne University Hospital, SE-20502 Malmö, Sweden, oskar.hansson@med.lu.se, phone +46 40 33 50 36, fax +46 40 33 56 57

Supplementary methods

1.1 Animals

In addition to *App*$^{NL-F/NL-F}$ and *App*$^{NL/NL}$ knock-in mice, male and female 3xTg mice (3-24 months, n = 42) were used for assessment of Aβ in CSF and brain tissue sections. Together with endogenous expression of M146V-mutated human presenilin 1 (PS1), these mice overexpress human APP with the Swedish (KM670/671NL) mutation as well as human MAPT$_{(4R0N)}$ with the P301L mutation under the control of the mouse Thy1 promoter element. This results in cerebral accumulation of intracellular Aβ from 3 months of age and deposition of extracellular amyloid plaques from 6 months of age (1), although phenotypic drift in the development of Aβ pathology have been reported in various colonies (2).

1.2 Histology and immunohistochemistry

30 µm thick free-floating sagittal brain sections from 3xTg mice were washed 3 × 10 minutes in TBS, treated 8 minutes with 88% formic acid (FA), permeabilize 3 x 10 minutes in TBS containing 0.25% Triton X-100 (TBSX), and blocked 1 hour in TBSX containing 5% normal donkey serum (NDS). The sections were then incubated with anti-Aβ primary antibody (MOAB-2, Millipore) diluted 1:1000 in TBSX containing 2.5% NDS overnight at 4°C. Following overnight incubation, the sections were washed 3 x 10 minutes in TBSX, incubated with appropriate Alexa-fluorophore-conjugated secondary antibody diluted 1:200 in TBSX containing 2.5% NDS, washed 3 x 10 minutes in TBSX, mounted on glass slides and coverslipped with ProLong^{TM} Diamond Antifade Mountant according to the recommendations from the manufacturer.

1.3 Image acquisition and analysis

Fluorescence images of whole brain sections from 3xTg mice were acquired using a 10x objective lens on the Operetta® CLS^{TM} High Content Analysis System (PerkinElmer). Cortex from one brain section per mouse were manually segmented and the area covered by Aβ-positive staining was quantified using the Fiji software by applying an automated local threshold that was maintained for all images analyzed.

References

1. Oddo S, Caccamo A, Shepherd JD, Murphy MP, Golde TE, Kayed R, et al. Triple-transgenic model of Alzheimer's disease with plaques and tangles: intracellular Abeta and synaptic dysfunction. Neuron. 2003;39(3):409-21.
2. Javonillo DI, Tran KM, Phan J, Hingco E, Kramar EA, da Cunha C, et al. Systematic Phenotyping and Characterization of the 3xTg-AD Mouse Model of Alzheimer's Disease. Front Neurosci. 2021;15:785276.

Supplementary figures

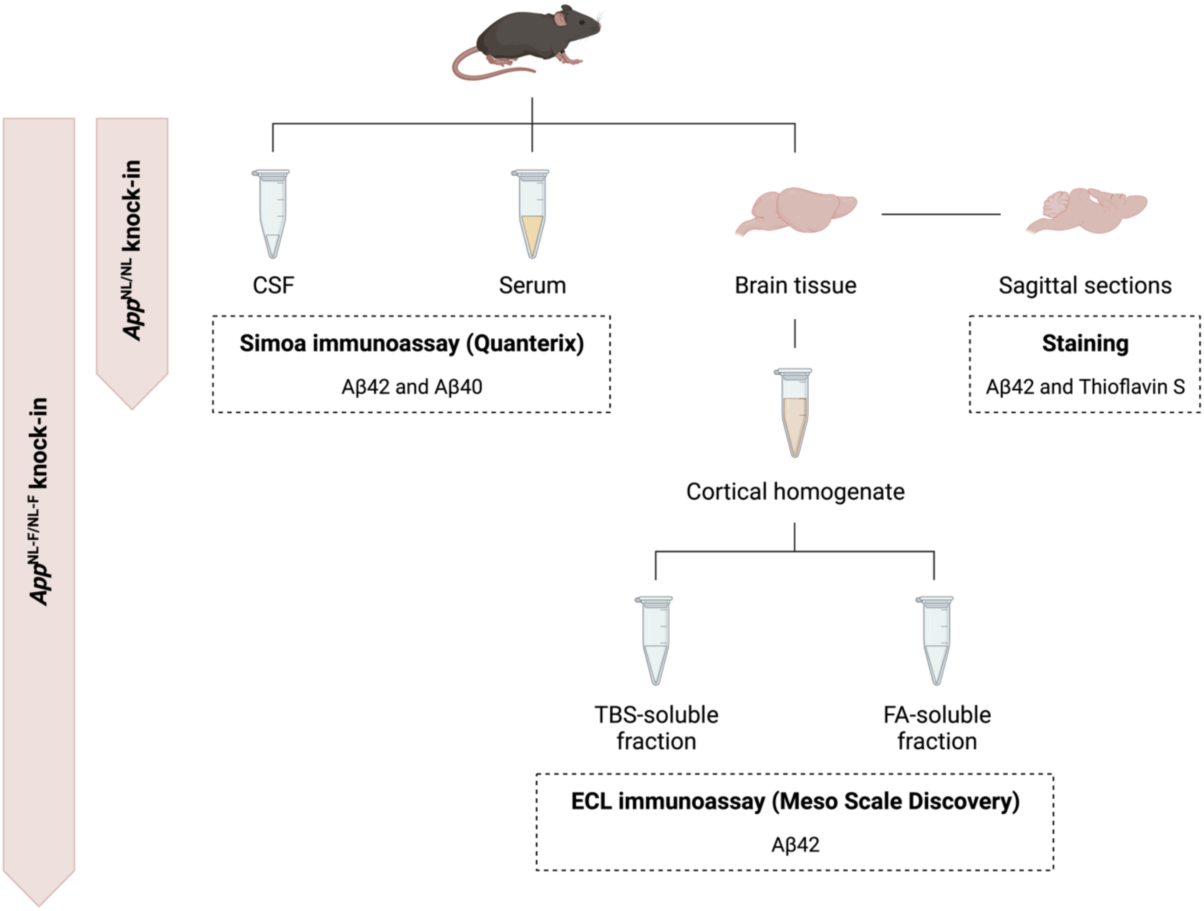

Figure S1: Experimental design
The illustration shows the experimental design of the study (created with BioRender.com). Abbreviations: Aβ, amyloid beta; APP, amyloid precursor protein; CSF, cerebrospinal fluid; ECL, electrochemiluminescence; FA, formic acid; Simoa, single molecule array; TBS, tris-buffered saline.

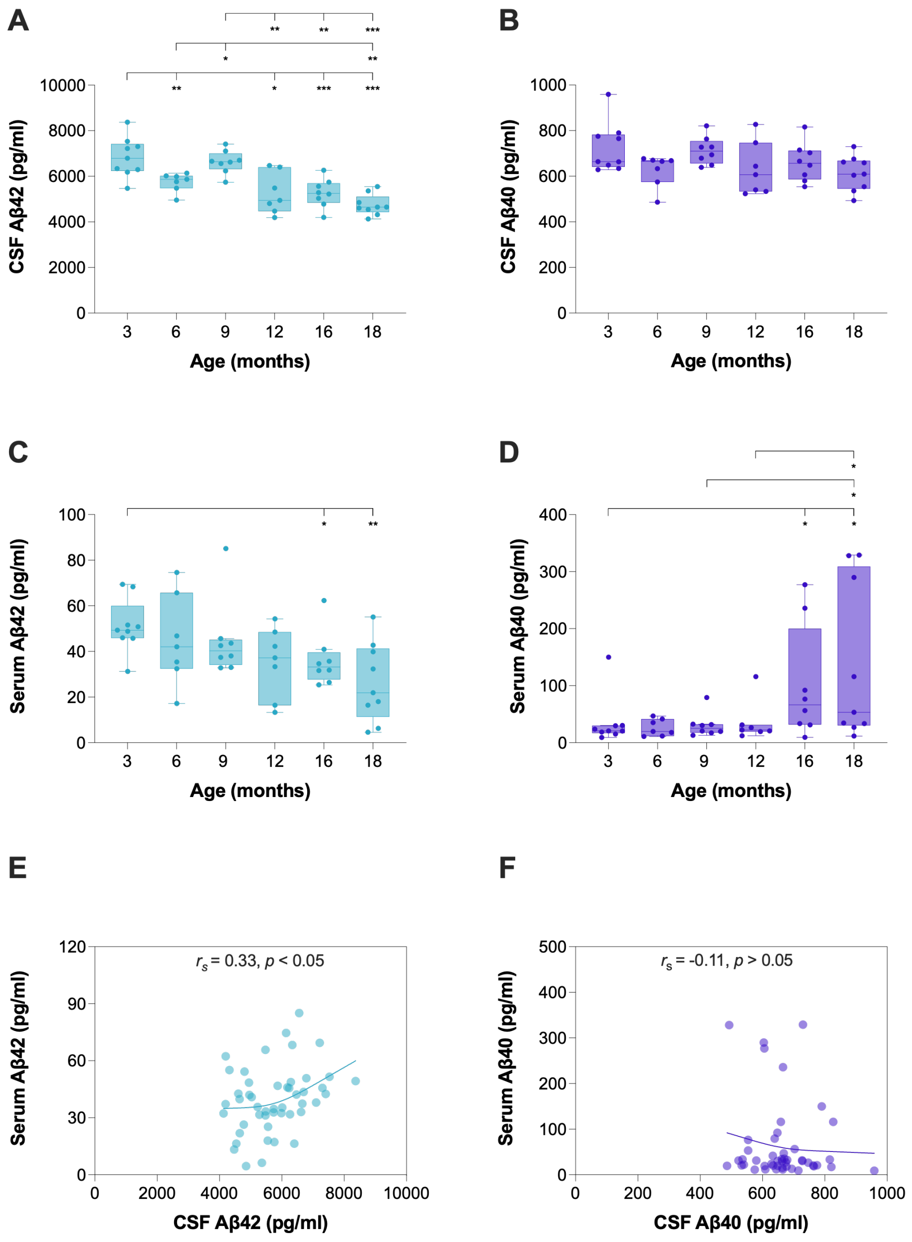

Figure S2: CSF and serum Aβ42 and Aβ40 in *App*$^{NL\text{-}F/NL\text{-}F}$ knock-in mice

CSF and serum Aβ42 and Aβ40 were measured in 3 (n = 9)-, 6 (n = 7)-, 9 (n = 8)-, 12 (n = 7)-, 16 (n = 8)-, and 18 (n = 9)-months-old *App*$^{NL\text{-}F/NL\text{-}F}$ knock-in mice. (**A**) CSF Aβ42 showed a steady significant reduction from 12 months of age while (**B**) no age-dependent effect on CSF Aβ40 was found. (**C**) Serum Aβ42 was significantly reduced from 16 months of age while (**D**) serum Aβ40 was significantly increased from this time point. (**E**) Serum Aβ42 correlated significantly positive with CSF Aβ42 while (**F**) no correlation between serum and CSF Aβ40 was found. Data is presented as median and IQR. Whiskers represent data within 1.5IQR of the lower and upper quartiles. For comparison between groups, statistical analyses were perform using the Kruskal-Wallis H test followed by the Mann-Whitney U test for *post hoc* group comparisons (*$p < 0.05$, **$p < 0.01$, ***$p < 0.001$). Correlation analyses were performed using Spearman's rank-ordered correlation coefficient. Abbreviations: Aβ, amyloid beta; CSF, cerebrospinal fluid; IQR, interquartile range.

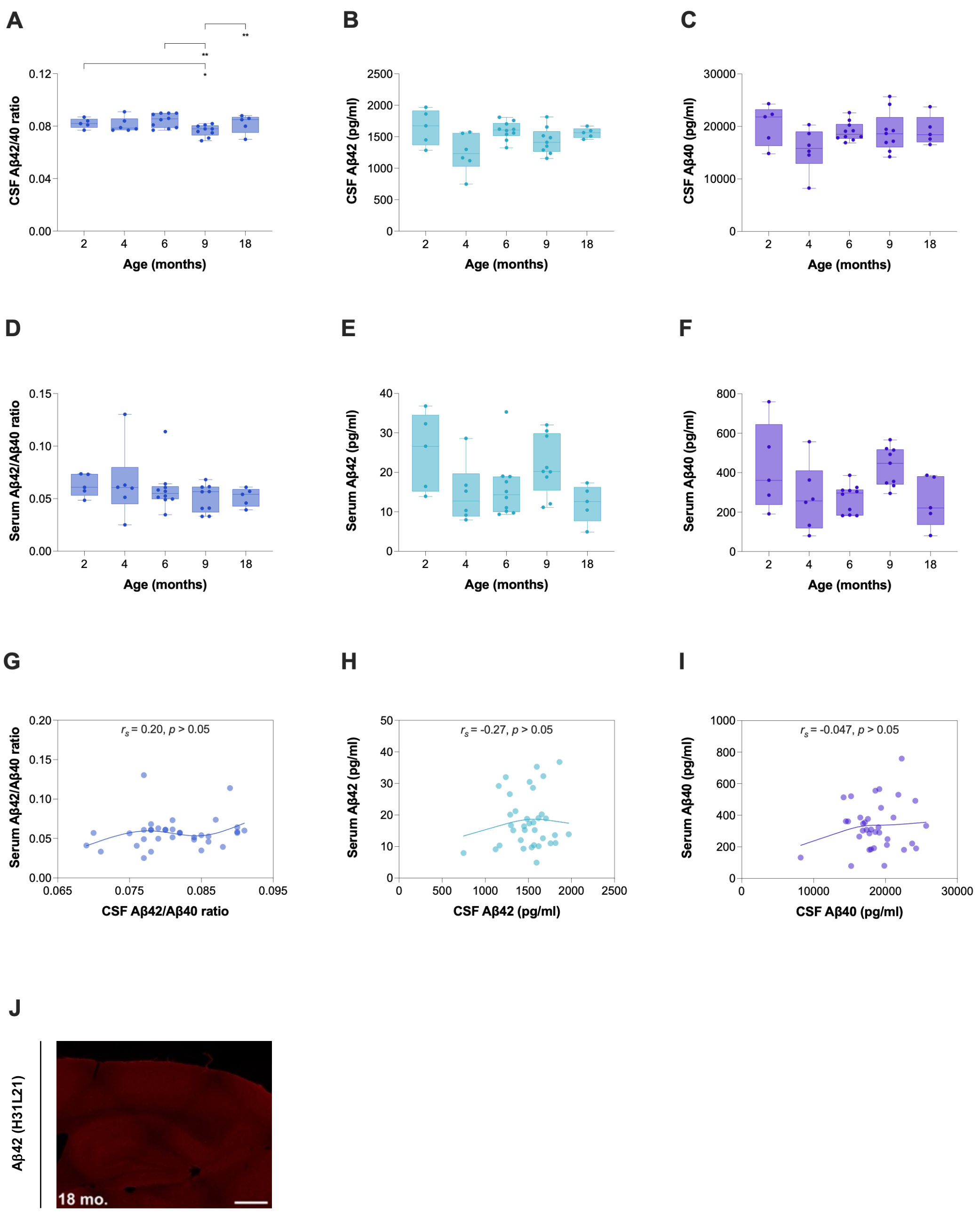

Figure S3: CSF and serum Aβ42/Aβ40 ratio, Aβ42, and Aβ40 in *App*$^{NL/NL}$ knock-in mice
CSF and serum Aβ42 and Aβ40 were measured in 2 (n = 5)-, 4 (n = 6)-, 6 (n = 10)-, 9 (n = 9)-, and 18 (n = 5)-months-old *App*$^{NL/NL}$ knock-in mice. (**A**) Some fluctuations in the CSF Aβ42/Aβ40 ratio was found with age although no age-dependent effect on **(B)** CSF Aβ42 or **(C)** CSF Aβ40 was observed. No change in **(D)** the Aβ42/Aβ40 ratio, **(E)** Aβ42, or **(F)** Aβ40 in serum was found with age. There was no correlation between **(G)** the Aβ42/Aβ40 ratio, **(H)** Aβ42, or **(I)** Aβ40 in serum and corresponding measures in CSF. **(J)** *App*$^{NL/NL}$ knock-in mice did not show any apparent cerebral Aβ deposition with age. Data is presented as median and IQR. Whiskers represent data within 1.5IQR of the lower and upper quartiles. For comparison between groups, statistical analyses were perform using the Kruskal-Wallis H test followed by the Mann-Whitney U test for *post hoc* group comparisons (*$p < 0.05$, **$p < 0.01$). Correlation analyses were performed using Spearman's rank-ordered correlation coefficient. Scale bar: 500 µm. Abbreviations: Aβ, amyloid beta; CSF, cerebrospinal fluid; IQR, interquartile range.

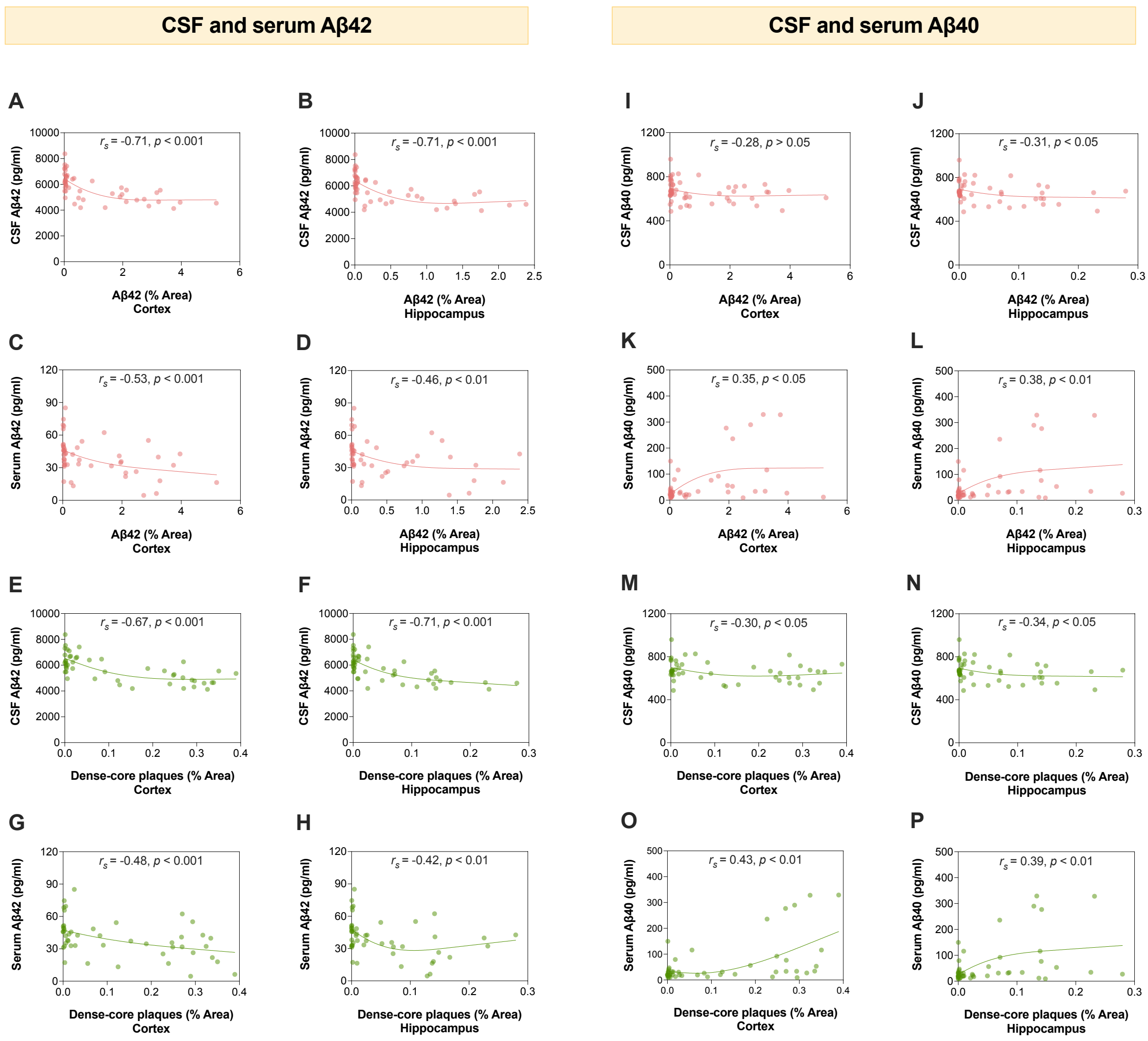

Figure S4: CSF and serum Aβ42 and Aβ40 and their associations with cerebral Aβ plaque burden in *App*$^{\text{NL-F/NL-F}}$ knock-in mice

In the whole study population, CSF and serum Aβ42 inversely correlated with **(A-D)** Aβ42 immunoreactivity and (**E-H**) thioflavin S-positive fibrillar dense-core plaques in cortex and hippocampus. (**I**) CSF Aβ40 did not correlate with Aβ42 immunoreactivity in cortex while **(J)** a weak negative correlation with corresponding measures in hippocampus as well as (**M-N**) thioflavin S-positive fibrillar dense-core plaques in hippocampus and cortex was found. A positive correlation was found between serum Aβ40 and (**K-L**) Aβ42 immunoreactivity as well as (**O-P**) thioflavin S-positive fibrillar dense-core plaques in cortex and hippocampus. Correlation analyses were performed using Spearman's rank-ordered correlation coefficient Abbreviations: Aβ, amyloid beta; CSF, cerebrospinal fluid.

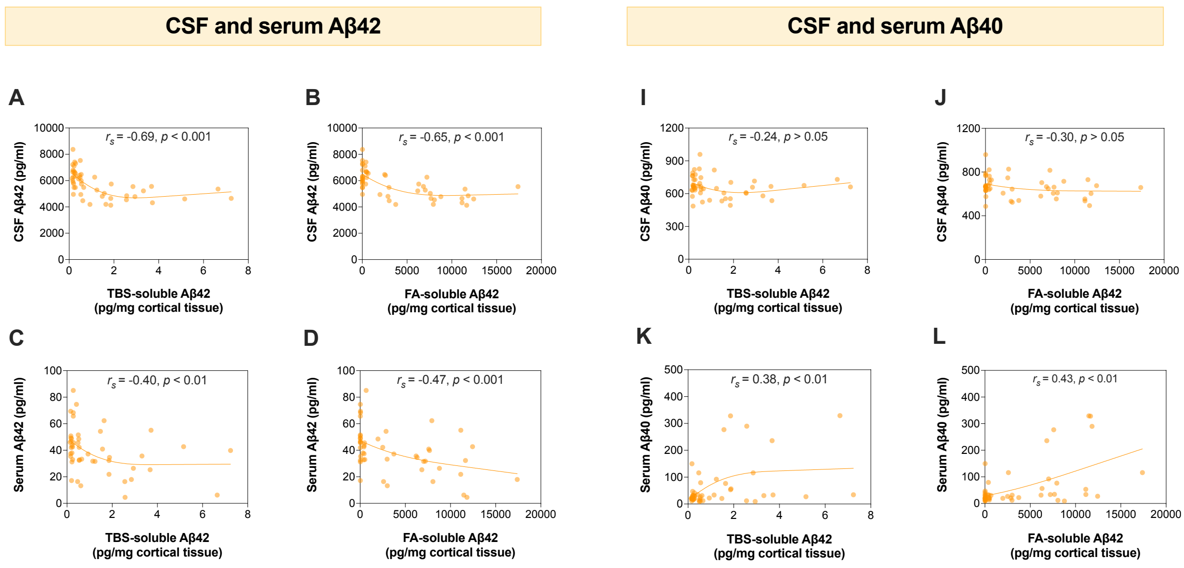

Figure S5: CSF and serum Aβ42 and Aβ40 and their associations with cortical TBS- and FA-soluble Aβ42 in *App*$^{NL-F/NL-F}$ knock-in mice

In the whole study population, CSF and serum Aβ42 inversely correlated with Aβ42 in the (**A and C**) TBS-soluble fraction as well as the (**B and D**) FA-soluble fraction prepared from cortical brain tissue homogenates. CSF Aβ40 did not correlate with Aβ42 in either the **(A)** TBS-soluble fraction or the **(B)** FA-soluble fraction. A positive correlation was found between serum Aβ40 and **(C)** TBS-soluble Aβ42 as well as **(D)** FA-soluble Aβ42. Correlation analyses were performed using Spearman's rank-ordered correlation coefficient. Abbreviations: Aβ, amyloid beta; CSF, cerebrospinal fluid; FA, formic acid; TBS, tris-buffered saline.

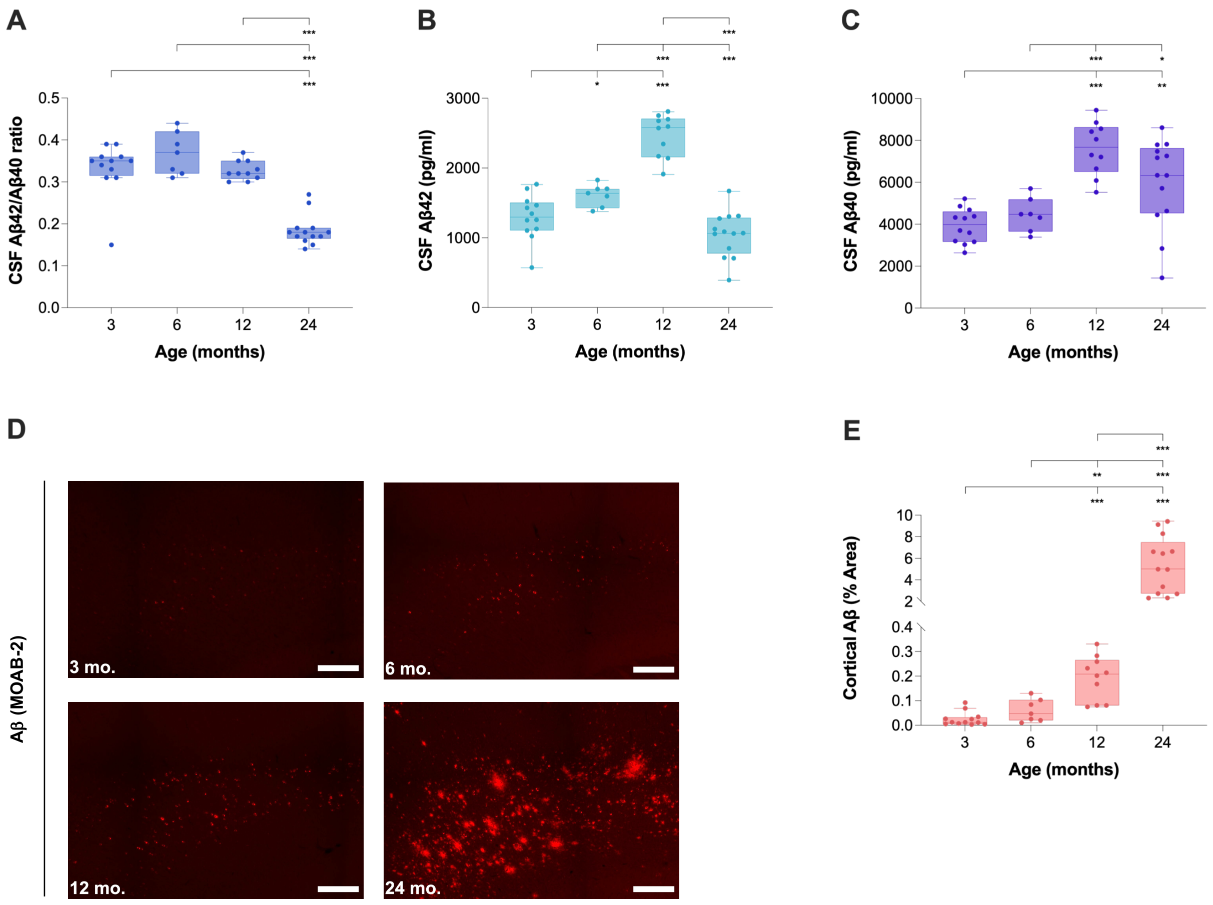

Figure S6: CSF Aβ42/Aβ40 ratio, Aβ42, Aβ40, and their relation to cerebral Aβ deposition in 3xTg mice

CSF Aβ42 and Aβ40 were measured in 3 (n = 12), 6 (n = 7), 12 (n = 10), and 24 (n = 13) months old 3xTg mice. **(A)** The CSF Aβ42/Aβ40 ratio was significantly reduced at 24 months of age. **(B)** CSF Aβ42 was increased up to 12 months of age after which a significant reduction was observed. **(C)** CSF Aβ40 was increased up to 12 months of age whereafter no further significant change was found. **(D)** Representative images of cortical Aβ immunoreactivity showing accumulation of intracellular Aβ from 3 months and extracellular Aβ plaque deposition at 24 months. **(E)** Quantification of cortical Aβ immunoreactivity revealed a significantly higher Aβ burden with increased age. Data is presented as median and IQR. Whiskers represent data within 1.5IQR of the lower and upper quartiles. For comparison between groups, statistical analyses were perform using the Kruskal-Wallis H test followed by the Mann-Whitney U test for *post hoc* group comparisons (*$p < 0.05$, **$p < 0.01$, ***$p < 0.001$). Scale bars: 250 µm. Abbreviations: Aβ, amyloid beta; CSF, cerebrospinal fluid; IQ R, interquartile range.

Supplementary tables

Table S1: Cerebral Aβ plaque burden in App$^{NL-F/NL-F}$ knock-in mice

	3 months	6 months	9 months	12 months	16 months	18 months	Kruskal-Wallis H test *p*-value
**Aβ42 immunoreactivity cortex (%Area)**	0.026 (0.019-0.035)	0.035 (0.024-0.054)	0.078 (0.057-0.10)$^{b, d}$	0.49 (0.34-0.56)$^{b, e, g}$	1.93 (1.46-2.09)$^{b, e, g, i}$	3.22 (2.81-3.85)$^{b, e, g, i, l}$	< 0.001
**Aβ42 immunoreactivity hippocampus (%Area)**	0.0053 (0.002-0.026)	0.0077 (0.0046-0.14)	0.029 (0.014-0.035)^{c}	0.15 (0.14-0.22)$^{b, e, g}$	0.76 (0.49-0.92)$^{b, e, g, i}$	1.67 (1.33-1.96)$^{b, e, g, i, k}$	< 0.001
**Dense-core plaques cortex (%Area)**	0.0010 (0.00050-0.0020)	0.0032 (0.0030-0.0060)^{a}	0.018 (0.012-0.027)$^{b, e}$	0.091 (0.069-0.12)$^{b, e, g}$	0.26 (0.23-0.27)$^{b, e, g, i}$	0.32 (0.29-0.34)$^{b, e, g, i, k}$	< 0.001
**Dense-core plaques hippocampus (%Area)**	0.00032 (0-0.0010)	0.0014 (0.00024-0.0027)	0.0042 (0.00082-0.0096)^{a}	0.024 (0.0093-0.050)$^{b, e, f}$	0.079 (0.069-0.14)$^{b, e, g, h}$	0.14 (0.13-0.22)$^{b, e, g, i, j}$	< 0.001

Data is presented as median and IQR. For comparison between groups, statistical analyses were perform using the Kruskal-Wallis test followed by the Mann-Whitney U test for *post hoc* group comparisons. Abbreviations: Aβ, amyloid beta; CSF, cerebrospinal fluid.

^{a} $p < 0.01$ vs. 3 months, ^{b} $p < 0.001$ vs. 3 months, ^{c} $p < 0.05$ vs. 6 months, ^{d} $p < 0.01$ vs. 6 months, ^{e} $p < 0.001$ vs. 6 months, ^{f} $p < 0.05$ vs. 9 months, ^{g} $p < 0.001$ vs. 9 months, ^{h} $p < 0.01$ vs. 12 months, ^{i} $p < 0.001$ vs. 12 months, ^{j} $p < 0.05$ vs. 16 months, ^{k} < 0.01 vs. 16 months, ^{l} < 0.001 vs. 16 months

Table S2: Correlations between CSF or serum Aβ42 and cerebral Aβ pathology in *App*$^{NL\text{-}F/NL\text{-}F}$ knock-in mice

	**CSF Aβ42**	**Serum Aβ42**	**Meng's Z-test (*p*-value)**
**Cortical Aβ42 (%Area)**	r_s = -0.71	r_s = -0.53	0.13
**Hippocampal Aβ42 (%Area)**	**r_s = -0.71**	**r_s = -0.46**	**0.045**
**Cortical dense-core plaques (%Area)**	r_s = -0.67	r_s = -0.48	0.14
**Hippocampal dense-core plaques (%Area)**	**r_s = -0.71**	**r_s = -0.42**	**0.023**
**TBS-soluble Aβ42 (pg/mg cortical tissue)**	**r_s = -0.69**	**r_s = -0.40**	**0.027**
**FA-soluble Aβ42 (pg/mg cortical tissue)**	r_s = -0.65	r_s = -0.47	0.17

Correlation analyses were performed using Spearman's rank-ordered correlation coefficient. Differences between correlation coefficients were estimated using Mengs Z-test. Abbreviations: Aβ, amyloid beta; CSF, cerebrospinal fluid.
